# *PLoS Pathogens* at Three Years

**DOI:** 10.1371/journal.ppat.1000167

**Published:** 2008-09-26

**Authors:** Kasturi Haldar, Grant McFadden

**Affiliations:** 1 Department of Biological Sciences, University of Notre Dame, Notre Dame, Indiana, United States of America; 2 Department of Molecular Genetics and Microbiology, College of Medicine, University of Florida, Gainesville, Florida, United States of America

As *PLoS Pathogens* turns three, we are pleased to say that the publication is flourishing. A journal's overall health can be measured in numerous ways: high quality and breadth of the research; high citation counts of individual papers; solid submission numbers; rising impact factor; wide influence and robust discussion within the community; strong editorial leadership; rapid publishing speed; accessibility of research; and, in our case especially, an unwavering commitment to creativity and new facets of open-access (OA) publishing. In all of these areas, the journal has grown and gained strength in the past three years through the collaborative effort of the community, editorial board, and PLoS staff to produce a publication that is both innovative and integrative.

The crux of *PLoS Pathogens'* strength lies in its vision and commitment to publish “outstanding original articles that significantly advance the understanding of pathogens and how they interact with their host organisms” and to make those articles immediately and widely available. By publishing a broad range of topics, including viruses, bacteria, prions, yeast and fungi, and parasites, readers can cross-reference methodologies and findings across various disciplines that traditionally have been segregated into specialist journals. This provides not only a breadth of information but also unique interdisciplinary consultations among outstanding editors whose expertise crosses both pathogens research efforts and the various scientific communities.

As the journal completes its first three-year editorial term, we especially thank the editorial board for their willingness to make a sustained commitment to *PLoS Pathogens* and the quality of work we strive to publish. We also thank the pathogens community for their groundswell of support, as evidenced in a trend of record manuscript submissions each month. This support in turn drives the need to continue to build a diverse editorial board that is integrated with its members' imperative to publish research of the highest caliber with significance across pathogens.

*PLoS Pathogens* is committed to OA publishing, making all of our content freely available to read, download, and reuse. The business model for and opportunities available through OA publishing are still evolving, but as funding agencies and academic institutions around the world advance laws, mandate actions, and launch key initiatives to support full access to published scientific articles, *PLoS Pathogens'* reach will only continue to expand.

This year *PLoS Pathogens* also provides a unique outlet among pathogens research journals with its transition to a new Web publishing platform, known as Topaz. Accepted manuscripts are now published more rapidly, and the online functionality enables members of the community to interact with the science and each other through erudite discourse on the published articles. This discussion is overseen by our new team of Community Editors, who encourage and review responses to the published articles—fostering a new method of increasing the journal's utility, accessibility, value, and ultimate impact on the field.

Thus, in three years, *PLoS Pathogens* has established itself as a leading journal that is poised to grow further. We are proud of what has been achieved so far, in such a short period of time, and we welcome the opportunity to publish your best research on pathogens and their host interactions.

